# Supplementary material for: Prevalence of Undernutrition in Community-Dwelling Older Adults in The Netherlands: Application of the SNAQ65+ Screening Tool and GLIM Consensus Criteria
Source: Nutrients. 2023 Sep 9;15(18):3917. doi: 10.3390/nu15183917 (PMC10537760; doi:10.3390/nu15183917)
Supplement: Supplementary file 1 [file nutrients-15-03917-s001.zip › nutrients-2594548-supplementary.pdf]

Supplementary materials

Table S1. Characteristics of the LASA participants aged <80 years (n=846) and the DNFCS participants (n=607).

	**UNDERNUTRITION SCREENING SAMPLE (SNAQ^{65+})**		
	LASA sample	DNFCS sample	p-vaiue
N	846	607	
Sex female, n (%)	422 (50.7)	296 (48.8)	0.47
Age (years), mean±SD	72.1±4.1	70.6±3.9	**<0.001**
BMI (kg/m^2), mean ±SD	27.1±4.9	26.9±4.9	0.45
BMI, n (%)			0.92
Underweight	72 (8.6)	42 (6.9)	
Normal and overweight	587 (69.7)	443 (73.0)	
Obese	183 (21.7)	122 (20.1)	
Appetite, n (%)			**<0.001**
Good	769 (90.9)	589 (97.0)	
Poor	77 (9.1)	18 (3.0)	
≥4 kg involuntary weight loss, n (%)	36 (4.3)	25 (4.1)	0.90
Mobility limitations, n (%)			**<0.001**
No	586 (69.3)	582 (95.9)	
Yes	259 (30.7)	25 (4.1)	
Region, n (%)			0.30
West	379 (44.8)	282 (46.5)	
Northeast	274 (32.4)	174 (28.7)	
South	193 (22.8)	151 (24.9)	
Education, n (%)			**0.001**
Low	94 (11.1)	27 (4.5)	
Medium	275 (32.5)	197 (32.8)	
High	477 (56.4)	377 (62.7)	

Living situation, n (%)			0.56
Alone	219 (26.0)	166 (27.3)	
With others	624 (74.0)	441 (72.7)	

LASA= Longitudinal Aging Study Amsterdam; DNFCS= The Dutch National Food Consumption Survey; SNAQ^{65+}= Short Nutritional Assessment Questionnaire 65+; BMI=Body Mass Index.

Table S2. Characteristics of the LASA participants with complete data on all GLIM criteria (n=700) and those with no complete GLIM data (n=438).

	LASA sample		
	Complete GLIM data	No complete GLIM data	p-vaiue
N	700	438	
Sex male, n (%)	361 (51.6)	191 (43.6)	**0.01**
Age (years), mean±SD	74.9 (6.6)	76.2 (7.1)	**0.001**
BMI (kg/m^2), mean ±SD	26.7 (4.2)	27.6 (5.3)	**0.005**
BMI, n (%)			0.11
Underweight	56 (8.0)	38 (8.8)	
Normal and overweight	510 (72.9)	286 (66.4)	
Obese	134 (19.1)	107 (24.8)	
Appetite, n (%)			**<0.001**
Good	652 (93.1)	357 (81.5)	
Poor	48 (6.9)	81 (18.5)	
≥4 kg involuntary weight loss, n (%)	30 (4.3)	34 (7.8)	**0.01**
Mobility limitations, n (%)			**<0.001**
No	469 (67.0)	234 (53.5)	
Yes	231 (33.0)	203 (46.5)	
Region, n (%)			**0.05**
West	329 (47.0)	180 (41.1)	
Northeast	213 (30.4)	163 (37.2)	
South	158 (22.6)	95 (21.7)	
Education, n (%)			**0.001**
Low	75 (10.7)	64 (14.6)	
Medium	223 (31.9)	164 (37.4)	
High	402 (57.4)	210 (47.9)	
Living situation, n (%)			0.28
Alone	213 (30.6)	145 (33.7)	
With others	482 (69.4)	285 (66.3)	

Receiving formal home care, n (%)			**<0.001**
Yes	81 (11.6)	84 (19.2)	
No	619 (88.4)	354 (80.8)	
Self-rated health, n (%)			**0.007**
Good	478 (68.3)	265 (60.5)	
Poor	222 (31.7)	173 (39.5)	

LASA= Longitudinal Aging Study Amsterdam; GLIM= Global Leadership Initiative on Malnutrition; BMI=Body Mass Index.
